# Langerhans Cell Histiocytosis of Thyroid Gland in a Child: A Case Report and Literature Review

**DOI:** 10.5152/eurasianjmed.2021.20144

**Published:** 2021-06

**Authors:** Rashad Ismayilov, Anar Aliyev, Aziz Aliyev, Ilgar Hasanov

**Affiliations:** 1 Department of Nuclear Medicine, Azerbaijan National Center of Oncology, Baku, Azerbaijan; 2 Department of Head and Neck Surgery, Azerbaijan National Center of Oncology, Baku, Azerbaijan; 3 Department of Pathology, Azerbaijan National Center of Oncology, Baku, Azerbaijan

## Introduction

Langerhans cell histiocytosis (LCH) is a rare neoplastic disease of dendritic cells with indefinite etiology and pathogenesis.^1^ The incidence rate of the disease is 4.0–5.4 per 1 million individuals, and the mortality rate is about 3% in adults.^2^,^3^ LCH is often encountered in pediatric patients and can be observed as single-organ involvement or multisystemic disease. The disease affects almost every organ in the body, including bone, skin, lung, lymph nodes, hypothalamopituitary axis, liver, spleen, and other sites.^2^,^4^ Thyroid involvement in LCH is rare and usually seen in adults.^5^ When it occurs in children, it is often accompanied by multisystemic involvement.^6^,^7^ Because of its rarity, LCH with involvement of the thyroid gland can cause delays in diagnosis and misdiagnosis.

## Case Report

**Figure 1:**
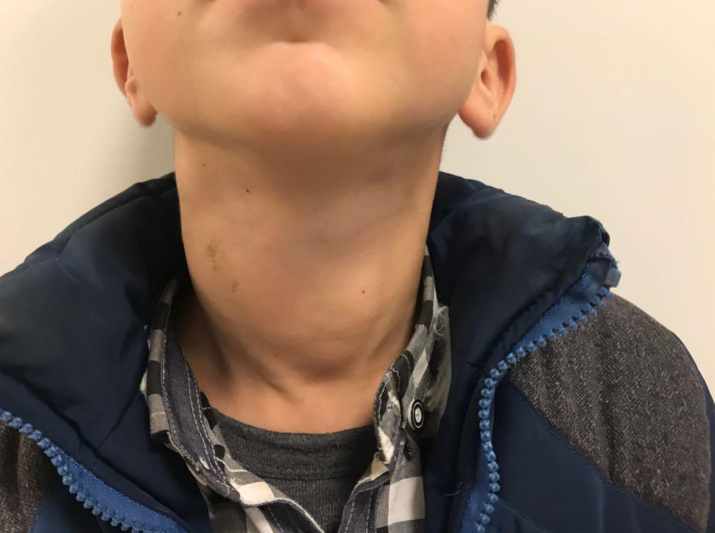
Physical examination. Anterior neck masses due to the thyroid and cervical lymph node involvement.

**Figure 2. a, b:**
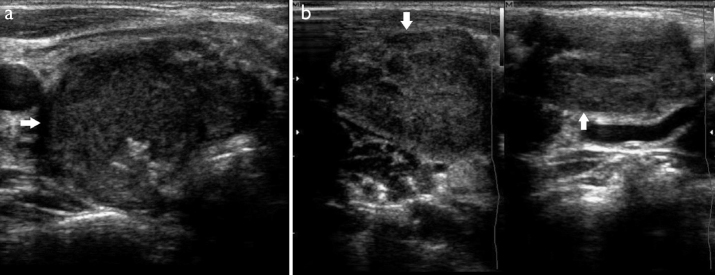
Neck ultrasonography. (a) Lymph nodes greater than 2 cm in the right lateral neck. (b) A 5.2 × 3 cm massive thyroid nodule with irregular borders covering a large part of the right lobe.

A 10-year-old boy was admitted to our clinic who had enlarged neck masses for 3 months. The patient had dyspnea, especially in sleep, and moderate dysphagia with solid foods. He had no fever, hoarseness, nausea, vomiting, or palpitations. Physical examination revealed a 5-cm non-tender mass over the anterior right neck and soft and matted cervical lymph nodes greater than 2 cm (Figure 1). Other system examinations were normal. The patient had no known illness and did not have a family history of thyroid disease and malignancy. Laboratory investigation showed that serum thyroid stimulating hormone was 11.8 mU/L (normal range, 0.4–4.2), free thyroxine was 5.53 pmol/L (normal range, 11.4–22.6), free triiodothyronine was 2.87 pmol/L (normal range, 3–6.7), serum thyroglobulin (Tg) was 393.6 ng/mL (normal range, 0–50), and anti-Tg and anti–thyroid peroxidase were negative. There was no abnormality in complete blood count or kidney and liver function tests. Erythrocyte sedimentation rate, lactate dehydrogenase, and urine analysis were normal. Ultrasonography (USG) of the neck revealed a 5.2 × 3 cm irregularly edged nodule with microcalcifications in the right lobe of the thyroid gland and hilus-erased lymphadenopathies greater than 2 cm in the right lateral neck (Figure 2). USG-guided fine-needle aspiration biopsy (FNAB) was performed on the thyroid nodule. The biopsy was reported as anaplastic epithelial tumor and/or histiocytosis: atypical lymphoid proliferation with no definitive discrimination. Afterward, the patient was investigated for systemic involvement. Computed tomography (CT) of the thorax demonstrated fine-walled bullae up to 6 cm in both lungs with a mid-to-upper lung zone predominance, no interstitial involvement, bilateral pneumothorax, and mediastinal lymphadenopathies up to 2.5 cm. There were no pathological findings on abdominal USG and CT. No evidence of disease involvement was found in the bone survey.

Considering the presence of compression symptoms, right lobectomy of the thyroid gland and excisional lymph node biopsy were performed. Pathology findings showed proliferation of Langerhans cells with nuclear grooves in a background of dispersed eosinophils. Immunohistochemical staining for CD1a and S100 were positive, and LCH was diagnosed. The *BRAF V600E* mutation was not detected by direct DNA sequencing.

The patient subsequently underwent chemotherapy, including vinblastine. Induction therapy was a dose of 6 mg/m^2^ every 7 days in combination with prednisone for 6 weeks, and maintenance was a dose of 6 mg/m^2^ every 3 weeks in combination with prednisone for 12 months. At follow-up 1 year later, there was no relapse in the thyroid gland. The largest of the lung bullae was smaller than 3 cm, and the total number of bullae decreased. Pneumothorax and pleural effusion were not observed. The size of cervical and mediastinal lymph nodes decreased.

Because the patient was under the age of 18, informed consent was obtained from the parents regarding use of the data and materials to be published.

## Discussion

The most common organ involvement in LCH is bones (80% of cases). It causes asymmetric osteolytic lesions in the bones and often affects the skull.^8^ Skin involvement is seen in approximately 33% of cases.^4^ The most common endocrinological abnormality in LCH is central diabetes insipidus.^9^ In approximately half of patients with LCH involving the thyroid, hypothalamopituitary abnormalities are observed, and usually these patients had central diabetes insipidus.^10^ In our case, no findings suggesting central diabetes insipidus were found.

Thyroid involvement in LCH usually manifests itself with nodular or diffuse enlargement of the gland (Table 1). Because of its rarity, it can be misinterpreted as benign goiters, undifferentiated carcinoma/anaplastic carcinoma, or lymphoma.^6^ It has also been shown that LCH of the thyroid accompanies primary thyroid malignancies at the same time.^11^

Different thyroid hormone conditions can be seen in patients with thyroid LCH. Most often, euthyroid and hypothyroid statuses are detected, but subclinical hypothyroidism and subclinical hyperthyroidism are also seen. In addition, anti-Tg and antimicrosomal antibodies rarely can be determined in cases of LCH involving the thyroid.^3^

USG and FNAB are the first methods used in the investigation of thyromegaly.^5^ Although FNAB of the thyroid is useful to establish a diagnosis, it can be confused with poorly differentiated or undifferentiated carcinoma.^7^,^12^ When FNAB is insufficient in diagnosis, the patient should be evaluated in terms of trucut/core biopsy. Fluorine-18-fluorodeoxyglucose positron emission tomography/CT (^18^F-FDG PET/CT) is not a common method in the diagnosis of thyroid LCH, but some reports have shown that it can be useful in diagnosis, staging, and evaluation of treatment. ^18^F-FDG PET/CT can also provide benefits in investigating other organ involvements.^13^ Because histopathological features may interfere with other diseases, CD1a and S100 positivity should be seen for a definite diagnosis.^5^,^14^ In some publications, it is stated that CD207 (Langerin) immunohistochemical staining will also help in the accurate diagnosis of LCH.^14^ When primary thyroid LCH is diagnosed, it is recommended to perform some tests, such as thoracic CT, bone scintigraphy, and abdominal USG, to determine the presence of multisystem involvement.^3^,^6^

There are no specific guidelines for the management of patients with LCH in the thyroid. Although isolated thyroid LCH can only be treated with thyroidectomy, systemic treatment is preferred in cases with multisystemic involvement.^4^,^5^ For disseminated aggressive disease, various chemotherapeutic agents can be used, including glucocorticoids, vinblastine, etoposide, methotrexate, doxorubicin, and cyclophosphamide. Because of the frequency of occurrences, a majority of adults underwent surgery alone; however, the majority of children underwent the combination of surgery and chemotherapy for thyroid LCH treatment.^3^,^7^

Consequently, although thyroid LCH is rarely seen, it should be kept in mind in patients with goiter. In some patients, multisystemic involvement may be overlooked and cause delays in diagnosis and treatment. In patients who cannot be diagnosed by FNAB and have compression symptoms, surgery may be useful in definitive diagnosis and treatment.

## Figures and Tables

**Table 1:** LCH Cases Presented with Thyroid Gland Involvement Published in the English Literature in the Last 5 Years

Year	Author	Age, years	G	Site	TSH, μIU/mL	fT4, pmol/L	fT3, pmol/L	Anti-TPO	Anti-Tg	BRAF mutations	Other thyroid pathology	her organ Otinvolvement	Treatment	Outcome
2016	Kuhn et al. (15)	73	F	Left	9.08	NR	NR	NR	NR	BRAF V600E (+)	Autoimmune thyroiditis	None	Total thyroidectomy	Alive
2016	AlZahrani et al. (16)	27	F	Bilateral	0.01	13.7	3.1	NR	NR	NR	PTC	Pituitary gland, lymph node, liver, spleen	Total thyroidectomy, RAI, prednisone, chemotherapy (NR)	Alive
2017	Wu et al. (17)	40	M	Bilateral	NR	NR	NR	NR	NR	NR	PTC	Lymph node, lung, liver, hypothalamus, palate	Right lobectomy, methotrexate, AraC	Alive
2019	Al Hamad et al. (18)	36	F	Bilateral	NR	NR	NR	NR	NR	BRAF V600E (+) in PTC and V600K (+) in LCH	PTC	Lymph node, skin	Total thyroidectomy, etoposide, prednisone	Alive
2019	Yokoyama et al. (8)	3	F	Bilateral	20.17	NR	NR	NR	NR	NR	None	Lung	Induction with AraC, VCR, and PSL (progression). Second course with ADR, VCR, CPM, PSL, and CyA	Alive
2019	He et al. (9)	3.5	M	Bilateral	87.8	10.36	2.92	Neg	Neg	BRAF V600E (−)	None	Lymph node, lung, liver, spleen, skin	PSL, JLSG-96 induction regimen, vemurafenib	Alive
2019	Zaidi et al. (19)	31	M	Right	NR	NR	NR	NR	NR	BRAF V600E (+) in PTC and/or LCH	PTC	Pituitary gland	Hemithyroidectomy, RAI, LCH-DAL HX-83 protocol	Alive
2020	Nacef et al. (20)	37	F	Right	NR	NR	NR	NR	NR	NR	None	Pituitary gland, bone, lymph node, lung	Total thyroidectomy, vinblastine, prednisone	Alive
2020	Ozisik et al. (11)	45	M	Bilateral	4.47	9.39	NR	NR	NR	BRAF V600E (+) in PTC and LCH	PTC	Pituitary gland, lymph node, gingiva	Total thyroidectomy, RAI	Alive
2020	Ozisik et al. (11)	58	M	Left	0.37	NR	NR	NR	NR	NR	PTC	None	Total thyroidectomy	
2020	Current case	10	M	Right	11.8	5.53	2.87	Neg	Neg	BRAF V600E (−)	None	Lymph node, Lung	Right lobectomy, prednisone, vinblastine	Alive

Abbreviations: ADR, doxorubicin; AraC, cytarabine; CPM, cyclophosphamide; CyA, cyclosporine A; F, female; fT3, free triiodothyronine; fT4, free thyroxine; G, gender; JLSG-96, Japan LCH study group-96; LCH, Langerhans cell histiocytosis; LCH-DAL HX-83, LCH Deutsche Arbeitsgemeinschaft für Leukaemieforschung Histocytosis X-83; M, male; Neg, negative; NR, not reported; PSL, prednisolone; PTC, papillary thyroid cancer; RAI, radioactive iodine treatment; Tg, thyroglobulin; TPO, thyroid peroxidase; TSH, thyroid stimulating hormone; VCR, vincristine.
